# Performance of the allometric power model in scaling from adult to paediatric antiretroviral dose in children at a Referral Hospital in Windhoek, Namibia

**DOI:** 10.4314/ahs.v22i3.47

**Published:** 2022-09

**Authors:** Bonifasius S Singu, Prisca Akpabio, Roger K Verbeeck

**Affiliations:** School of Pharmacy, Faculty of Health Sciences & Veterinary Medicine, University of Namibia

**Keywords:** Dose, weight, children, antiretrovirals, BSA, allometric scaling

## Abstract

**Background:**

World Health Organization (WHO) advocates use of weight bands in antiretroviral therapy (ART) guidelines. Allometric scaling could be a more reliable method because it uses a non-linear approach in relating dose to body weight. This study evaluates performance of the allometric ¾ power model in comparison to WHO weight band method in children receiving ART.

**Methods:**

Records of children receiving (ABC/3TC) + DTG were reviewed. Paediatric ABC/3TC dose was calculated from the adult dose using the allometric ¾ power model and compared to WHO weight band dose.

**Results:**

WHO weight band strategy grouped 50.6% of the children in the 25 kg category and therefore received the adult dose of ABC/3TC (600 mg/300 mg); only 1.1% received this dose with allometric scaling. Mean dose (3.8 tablets) for the WHO weight band dosing method was found to be significantly higher (p<0.0001) than for allometric scaling (1.5 tablets).

**Conclusions:**

WHO weight bands may result in the 25 kg weight category receiving a much higher dose leading to ADRs. Using allometric scaling, we recommend a weight band strategy that could improve paediatric ABC/3TC dosing.

## Introduction

The World Health Organization (WHO) guidelines for antiretroviral therapy (ART) in children (2019) state the preferred first-line regimen as abacavir (ABC) + lamivudine (3TC) + dolutegravir (DTG), and the alternative first-line regimen is ABC + 3TC + lopinavir/ritonavir (LPV/r). 1 Experts have advocated for the use of flexible solid oral fixed-dose combinations (FDC) of antiretroviral (ARV) drugs to encourage implementation of new paediatric treatment guidelines and improve adherence to ART. 2 The WHO advocates for the use of weight band dosing in both its guidelines for ART and tuberculosis (TB). 1,3 Scientific studies have recommended that targeted therapeutic and safe concentrations can be achieved with paediatric ABC/3TC fixed-dose combinations formulated as 30 mg/15mg per unit with a twice daily dose of 2, 3, 4, 5, 6 units for the respective weight bands of 4–5.9 kg, 6–9.9 kg, 10–13.9 kg, 14–19.9 kg, and 20–24.9 kg.^2^ Although therapeutic drug monitoring (TDM) is the best way to ensure the administered dose achieves target concentrations for safe and efficacious therapy, it is not practical for non-nucleoside reverse transcriptase inhibitors (NRTIs) such as ABC and 3TC because their activity is intracellular and measuring their concentrations in that space is both expensive and labour-intensive 4. LPV/r 40 mg/10 mg granule formulation is available for children and is administered with a target therapeutic range of 1–4 mg/L in both adults and paediatrics. 4,5 Recent and ongoing studies to simplify ART weight band dosing have led the WHO to recommend the use of the integrase strand transfer inhibitor (INSTI) dolutegravir (DTG) 50 mg dose for children with a body weight of 25 kg and above; formulated as a film-coated 10 or 25 mg tablet, DTG has become a mainstay antiretroviral (ARV) in combination with ABC/3TC in children of ages >6 years and weighing more than 15 kg due to its strong viral suppression, low viral resistance profile, and high therapeutic index with lesser drug-drug interactions as compared to LPV/r. 4. In the efforts to establish the most appropriate method for scaling adult drug doses to children some have proposed the use of allometric scaling instead of the traditional linear scaling methods such as body weight (BW) and body surface area (BSA) to allometric model 6,7,8.

The allometric power model uses nonlinear relationships and is a more reliable way to relate dose to body weight, it is generally expressed with the equation:


(1) $$Y=\alpha \times BW^{b}$$

Where: is the variable to be predicted, is an empirically derived constant, and is the exponent of correlation. Experimental findings have shown that variables that are time-related such as heart rate have an exponent of 0.25, and metabolic variables such as basal metabolic rate, including drug clearing enzyme activity (CL) corelate with body weight by an exponent of 0.75. 9,10. Therefore, drug clearance in children can be predicted from the adult value in the following way:

(2) $$CL_{\mathrm{child}}=CL_{\mathrm{adult}}\times \left(\frac{\mathrm{Weigh}t_{\mathrm{child}}\;\mathrm{in}\;\mathrm{kg}}{70}\right)0.75$$

Since the maintenance dose for steady-state concentrations depends on the plasma concentration and drug clearance, dose can be substituted for clearance 10,11:

(3) $$\mathrm{Dos}e_{\mathrm{child}}=\mathrm{Dos}e_{\mathrm{adult}}\times {\left(\frac{\mathrm{Weigh}t_{\mathrm{child}}\;\mathrm{in}\;\mathrm{kg}}{70}\right)}^{0.75}$$

Namibia follows the WHO guidelines for ART therapy. This study was carried out to evaluate the performance of the allometric ¾ power scaling model in comparison to the WHO weight band dosing method in dose determination for children receiving ABC/3TC-based ART at Katutura Intermediate Referral Hospital, Windhoek, Namibia.

## Methods

This was an analytic retrospective review of paediatric patient clinical records at the ART clinic based at Katutura Intermediate Referral Hospital (KIRH), Windhoek, Namibia. Convenience sampling was employed to include records of patients who were within the age range of 2–12 years on their most previous birthday and were currently receiving ABC/3TC-based ART therapy until the sample size was obtained. Patient data retrieved were those recorded for the most recent visit to the ART clinic; the following patient information were collected: date of birth, date of last visit, height, weight, sex, dose of ABC/3TC received.

For purpose of comparison, the paediatric ABC/3TC dose was determined using two methods: in the first method, weight-banded dose was determined using the current recommended guidelines for paediatric dosing of ARVs (Table 1), and in the second, paediatric ABC/3TC dose was calculated from the adult dose (600 mg/300 mg) by use of the allometric ¾ power model (equation 3):

**Table 1:** Paediatric ARV Dosage Chart

Drug	Formulation strength	Number of tablets/sachets by weight (once daily)					Strength of adult tablet	Number of tablets
		3–5.9 kg	6–9.9 kg	10–13.9 kg	14–19.9 kg	20–24.9 kg		25–34.9 kg
ABC/3TC	Dispersible tablet: 120mg/60mg	1	1.5	2	2.5	3	600 mg/ 300 mg	1
LPV/r	Granules: 40mg/10mg sachet	2 BD	3 BD	4 BD	5 BD	6 BD	-	-
DTG	Tablet: 50mg	-	-	-	-	1	50 mg	1

Namibia ART Guidelines 6^th^ Edition, 2019^12^

The paediatric dose calculated in mg by allometric power model was then converted to the closest equivalent dose in tablet numbers: 120 mg/60 mg (1 tablet), 150 mg/90 mg (1.5 tablets), 240 mg/120 mg (2 tablets), 300 mg/150 mg (2.5 tablets), 360 mg/180 mg (3 tablets), or 600 mg/300 mg (adult dose). These doses were then compared to those received by the patients when the WHO weight band strategy is used. To determine the new weight bands in table 3, Equation 3 was rearranged:

(4) Patient weight in kg = 70(Paediatric dose (mg)Adult dose (mg))10.75

**Table 3:** Recommended paediatric ABC/3TC weight band dosing table based on allometric scaling (calculated using equation 4)

Body weight (kg)	Number of tablets (120mg/60mg)
<8.2	1
8.2–14.1	1.5
14.2–20.6	2
20.7–27.8	2.5
27.9–35.4	3
>35.5	1 adult dose tablet (600mg/300mg)

Since the data was found to not be normally distributed, a Wilcoxon rank sum test with continuity correction (Mann-Whitney test) was performed using Excel (Microsoft Corp., WA, USA) to compare the difference in the mean dose using the two methods. A p value < 0.05 was considered statistically significant.

Ethical clearance and approval to conduct this study was obtained from the Ministry of Health and Social Services, Namibia. As this study was a review of patient records, informed consent from parents/guardians was not necessary. Patient names were not included as part of the data collected for this study in order to protect the identity of patients whose records were accessed.

## Results

Out of a total population of 183 children receiving ARV therapy at KIRH ART clinic, records of 89 (44.2%) paediatric patients (40 male and 49 female) were suitable and included in this study. Data collected were for patients who visited the clinic between May and July 2020. All children received their dose based on a FDC tablet formulated as ABC/3TC (120 mg/60 mg) together with either LPV/r (40 mg/10 mg granules or 80 mg/20 mg per mL syrup) for children with body weight <20 kg or dolutegravir 50 mg tablet for children who weighed >20 kg. The 89 children included in this study had a median age and weight of 9 years (range of 2–12) and 25.0 kg (range of 9.6–58.8), respectively. Table 2 breaks down these demographics further.

**Table 2:** Ages and body weights of children on ABC/3TC (120mg/60mg) FDC (n=89)

Age (years)	Count (n)	Cumulative frequency (%)	Weight (kg) Mean (range)
2	4	4.49	12.0 (9.6–14.5)
3	4	8.99	13.4 (12.3–14.9)
4	2	11.24	12.9 (11.7–14.2)
5	3	14.60	16.5 (14.3–17.6)
6	4	19.10	18.8 (15.1–23.1)
7	9	29.21	19.8 (15.0–29.0)
8	11	41.57	24.0 (18.1–31.3)
9	9	51.69	23.5 (19.8–28.2)
10	11	64.04	28.3 (22.3–41.5)
11	15	80.90	29.4 (21.8–35.0)
12	17	100.00	31.9 (23.7–58.8)

**Figure 1:**
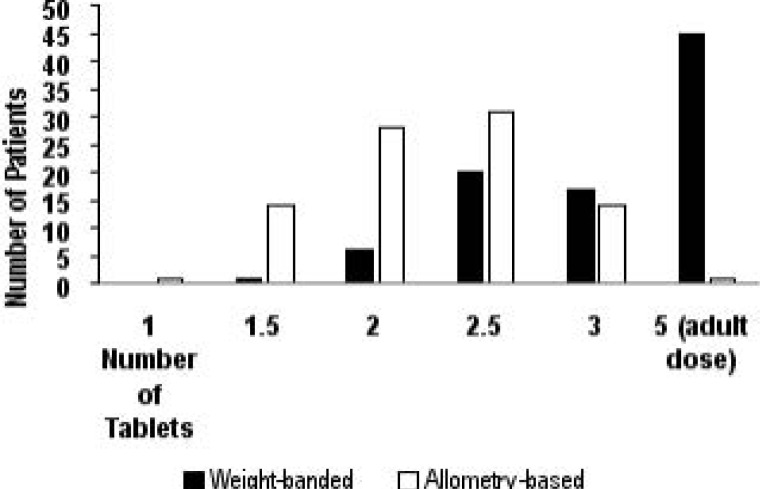
Distribution of paediatric patients when ABC/3TC dose is determined by weight-banding as recommended by ARV guidelines of 2019 (shaded black) as compared to when ABC/3TC dose is determined by allometric scaling (white). 1 tablet = ABC/3TC 120 mg/60 mg; adult dose = ABC/3TC (600 mg/300 mg). Children in the 25 kg weight band mostly ended up receiving the adult dose.

When using weight-banded dosing, 45 (50.6%) of the children were found to be receiving the adult dose of ABC/3TC (600 mg/300 mg) as compared to only 1 child (1.1%) when allometric scaling is applied (figure 1).

A Mann-Whitney test of the dose (as number of tablets) to compare the two methods found the mean±SD tablet dose for weight-banding (3.8±1.23) and allometric scaling (2.9±0.65) strategies to be significantly different (p<0.0001). Age and body weight were highly correlated (r = 0.78, r2 = 0.61, and p<0.0001).

## Discussion

As in virtually all other cases, drugs used in paediatric ART are developed with adults as subjects during clinical trials 12. Ethical restrictions limit the involvement of children in clinical trials for dose determination studies, and this leaves no other option but for their dosage regimens to be extrapolated from those of adults 12, 13. In clinical practice, linear correlation of dose with demographics such as age, body surface area (BSA), and weight have been the popular methods for scaling from the adult to the paediatric dose even when these methods have been known to be inaccurate 12. Allometric scaling is regarded to be a more reliable method because it uses a non-linear approach to relate dose to body weight which makes paediatric dosing recommendations effective and much safer 14.

In this study we assessed whether there would be a difference in dose for paediatric patients receiving ABC/3TC when the recommended dosing strategy in the ART guidelines (2019) of weight-banding is compared with the allometric scaling method. We found that the weight band method resulted in over half (50.6%) of the children with an age range of 7–12 years receiving the highest dose/adult dose of ABC/3TC (600 mg/300 mg) on the paediatric dosing chart since they were in the 25 kg body weight category (range = 25.0–58.8 kg); while allometric scaling resulted in only 1 patient (out of 89) who received the adult dose. In addition, when weight-banding is used the distribution of patients is skewed to the left with more patients receiving doses on the higher end; in contrast, and of note, the allometric strategy resulted in a gaussian distribution having fewer patients distributed on either ends of the dosing spectrum. Consequently, the mean dose was higher in the weight-banding strategy than when allometrically scaled.

In order to encourage public health approach and increase access of children to ART, the WHO introduced weight-band dosing in the effort to simplify the use of mg/m2 and mg/kg methods which require the involvement calculations by the prescriber/pharmacist 15,16. However, the main disadvantage of weight-band dosing is that the children on the higher and lower ends of one weight band are likely to receive either a higher or lower dose which may lead to toxicity or ineffective treatment.^4^. A study in South Africa reported that pyrazinamide doses result in different levels of exposure when patients are given the drug in different weight bands according to the WHO guidelines on TB therapy; the patients in the lower weight bands were found to have a lower and subtherapeutic exposure (AUC), therefore adjustment to the dose for each weight band was recommended. 17 The variation in paediatric weight for age patterns in different geographical regions of the world due to factors such as diet and genetics are important known causes of many children receiving total daily doses that are either too high or too low. 18,19.

Drug elimination is greatly influenced by maturation of metabolic capacity which is altogether unrelated to body weight because different enzyme systems are fully functional at different life stages, with some enzymes having peak activity in paediatric age group and waning or even completely disappearing in adulthood, and vice-versa.^14^,^20^,^21^. Development of metabolic pathways in children and adults can therefore not be accurately related by body weight differences because it may simply lead to subtherapeutic exposure in children 14. A study that compared the performance of BW, BSA, and BW0.75 models in scaling of adult dose to the paediatric using the British National Formulary for children 2006 (BNFC) found that BW0.75 and BSA gave better results than BW for children 7–12 years old; the BW model was also prone to give a lower dose 10. Total drug clearance may depend on glomerular filtration rate, liver blood flow, and the intrinsic ability of the liver enzymes to clear the drug which may vary both with developmental growth and with pathophysiology 22. Use of allometric scaling is believed to yield the most accurate drug exposure results in children because it reduces the error in predicting drug clearance in children, which under steady state conditions is the determinant of systemic exposure. 7,12,14,23. In conclusion, the allometric ¾ power model for scaling from the adult to the paediatric dose results in a normal distribution of patients with respect to dose. The mean dose was significantly higher for weight band dosing than for the allometry model. Furthermore, the results from this study demonstrate that dosing children according to WHO weight bands results in patients who were grouped in the 25 kg weight category receiving a much higher dose. For better dosing of children with a weight of 25 kg, we recommend for the WHO weight bands for ABC/3TC to be restructured as demonstrated in table 3. Furthermore, larger studies should be conducted comparing the adverse effects, tolerability, adherence, and clinical outcomes when the two methods are used.

The limitation of this study was that it was conducted at only one center for paediatric ART management based in only one country. The sample size may not be large enough for the results to be generalised to other paediatric populations in other countries.
